# Complete Genome Sequence of Actinomyces oris Strain K20, Isolated from an Oral Apical Lesion

**DOI:** 10.1128/mra.00541-22

**Published:** 2022-07-25

**Authors:** Takayuki Nambu, Chiho Mashimo, Hugo Maruyama, Makoto Taniguchi, Yao Huang, Hiroki Takigawa, Wenyan Kang, Qianqian Yan, Lei Zhang, Lin Yang, Kazuya Takahashi, Toshinori Okinaga

**Affiliations:** a Department of Bacteriology, Osaka Dental University, Hirakata, Osaka, Japan; b Genome-Lead Co., Ltd., Takamatsu, Kagawa, Japan; c Graduate School of Dentistry (Geriatric Dentistry), Osaka Dental University, Hirakata, Osaka, Japan; d Graduate School of Dentistry (Bacteriology), Osaka Dental University, Hirakata, Osaka, Japan; e Department of Geriatric Dentistry, Osaka Dental University, Hirakata, Osaka, Japan; University of Rochester School of Medicine and Dentistry

## Abstract

Actinomyces oris strain K20 was isolated from oral apical lesions. Here, we report the complete circular genome sequence of this strain, obtained by means of hybrid assembly using two next-generation sequencing datasets. The strain has a 3.1-Mb genome with 2,636 coding sequences.

## ANNOUNCEMENT

*Actinomyces* are ubiquitous in soil as well as human and animal microbiota (1, 2). Some *Actinomyces* species are well-known members of the oral microbiota (3). Actinomyces oris is a representative species of oral *Actinomyces* that adheres to tooth surfaces and other oral bacteria via specific fimbriae (4, 5). The isolation of *A. oris* from actinomycotic lesions, apical root abscesses, root canal infections, and peri-implantitis suggests its involvement in these diseases (1).

*A. oris* strain K20 was previously isolated in our laboratory as a dominant organism from apical abscess foci (6, 7). We previously attempted to sequence its genome to clarify the genetic background for biofilm formation. However, a complete genome sequence was not obtained because of its high G+C content (7). In this study, we attempted to sequence the complete genome of this strain using both long- and short-read sequences. The study was approved by the Ethics Committee of Osaka Dental University (Osaka, Japan; approval number 060641), and the study participant provided written informed consent.

The strain was cultured overnight in heart infusion broth (BD Difco) at 37°C under aerobic conditions. Genomic DNA (gDNA) was extracted from the culture medium of this strain using the MagAttract high-molecular-weight (HMW) DNA kit (Qiagen Inc., CA, USA). The obtained gDNA was subjected to long- and short-read sequencing. Long-read sequencing was performed using the GridION X5 sequencing platform (Oxford Nanopore Technologies [ONT], UK); 1.0 μg unfragmented gDNA was used for library construction using a ligation sequencing kit (SQK-LSK109; ONT). The prepared library was applied to an R9.4.1 flow cell (FLO-MIN106; ONT). The base calling of long-read sequences using Dogfish v.0.9.6-3 generated 114,245 reads (1.1 Gb), with an *N*_50_ value of 34,514 bp during a 10-h runtime. After quality trimming (average Phred quality value, >8.0) using NanoFilt v.2.3.0 (8), a total of 56,255 reads (748 Mb) were generated, with an *N*_50_ value of 35,974 bp. For short-read sequencing, 500 ng gDNA was fragmented to produce 300- to 350-bp-long fragments for library construction using the Nextera DNA Flex library prep kit (Illumina), following the manufacturer’s standard protocol. Paired-end (2 × 156-bp) reads were obtained using a MiSeq instrument (Illumina). The raw sequencing data (1,217,657 reads; average read length, 154.4 bp) were processed using the FASTQ preprocessing program fastp v.0.19.5 (qualified Phred quality score, 20) (9) to trim adapters and low-quality data, yielding 1.19 million short reads with an average length of 149.8 bp.

For complete *de novo* genome assembly, the remaining long- and short-read data were processed using the Unicycler v.0.4.4 pipeline (10). Assembly polishing, circularization, and rotation were performed using Unicycler. The polished circular assemblies were visualized using Bandage v.0.8.1 (11). BlobTools (12) was used to assess the presence of contaminant DNA. The contig sequence obtained was polished twice using Pilon v.1.23 (13) until no errors were detected; 13 regions were corrected. Default parameters were used for all software unless otherwise specified. The resulting complete genome consists of 3,119,201 bp and exhibits a G+C content of 68.3% (coverage, 114×). Automatic annotation performed using the annotation pipeline DFAST v.1.1.6 (14), provided by DDBJ (https://dfast.ddbj.nig.ac.jp/), predicted 2,636 coding sequences, 9 rRNA genes, and 52 tRNA genes.

### Data availability.

The complete genome sequence of *A. oris* strain K20 has been deposited at DDBJ/EMBL/GenBank under the accession number AP025590. The associated BioProject and BioSample accession numbers are PRJDB13022 and SAMD00442649, respectively. The SRA accession numbers are DRR351395 (Illumina) and DRR351396 (Nanopore).
